# Lipids, curvature, and nano-medicine*

**DOI:** 10.1002/ejlt.201100050

**Published:** 2011-10

**Authors:** Ole G Mouritsen

**Affiliations:** MEMPHYS – Center for Biomembrane Physics, Department of Physics and Chemistry, University of Southern DenmarkCampusvej, Odense M, Denmark

**Keywords:** Curvature, Drug delivery, Lipid shape, Liposome, Nano-medicine

## Abstract

The physical properties of the lamellar lipid-bilayer component of biological membranes are controlled by a host of thermodynamic forces leading to overall tensionless bilayers with a conspicuous lateral pressure profile and build-in curvature-stress instabilities that may be released locally or globally in terms of morphological changes. In particular, the average molecular shape and the propensity of the different lipid and protein species for forming non-lamellar and curved structures are a source of structural transitions and control of biological function. The effects of different lipids, sterols, and proteins on membrane structure are discussed and it is shown how one can take advantage of the curvature-stress modulations brought about by specific molecular agents, such as fatty acids, lysolipids, and other amphiphilic solutes, to construct intelligent drug-delivery systems that function by enzymatic triggering via curvature.

**Practical applications:** The simple concept of lipid molecular shape and how it impacts on the structure of lipid aggregates, in particular the curvature and curvature stress in lipid bilayers and liposomes, can be exploited to construct liposome-based drug-delivery systems, e.g., for use as nano-medicine in cancer therapy. Non-lamellar-forming lysolipids and fatty acids, some of which may be designed to be prodrugs, can be created by phospholipase action in diseased tissues thereby providing for targeted drug release and proliferation of molecular entities with conical shape that break down the permeability barrier of the target cells and may hence enhance efficacy.

## 1 Introduction

Our picture of lipid membranes has come a long way since Gorter and Grendel in 1925 formulated the lipid-bilayer hypothesis 1–3. Most textbook models of membranes are still based on the celebrated fluid-mosaic Singer–Nicolson model from 1972 4–6, although we have in recent years seen significant amendments to this model, not least fuelled by the finding of lipid membrane domains in both model membranes and cells 7–12 and the subsequent “raft rush” 13–17. The science of *lipidology* has now become an established discipline, acknowledging that lipids organize in space and time and display emergent physico-chemical properties that are beyond the chemical nature of the individual molecules and which collectively control membrane function 8.

Recently, *lipidomics* has followed as a new science in the omics-sequel, characterized by an explosion in detailed data for lipid profiles of tissues, cells, and subcellular components 18. The focus is now swinging toward enumerating individual lipid species, determining their identity, and quantitating their amount. Time is ripe to marry the two disciplines, both in order to take lipidomics beyond the stage of “stamp collection” 8 and in order to incorporate into the lipidology approach the new knowledge about the individual lipid species. I will illustrate my viewpoint in the present mini-review by discussing the use of the old concepts of lipid shape and membrane curvature in the context of trans-membrane structure, membrane permeability, and enzymatic action. I will go on to demonstrate how insights into lipid shape and membrane curvature can be translated into technological applications within nano-medicine and drug delivery mediated by liposomes. In particular I will show how lipids may serve as prodrugs, pro-enhancers, and pro-permeabilizers within liposomal drug delivery in cancer therapy 19, 20.

**Figure 1:**
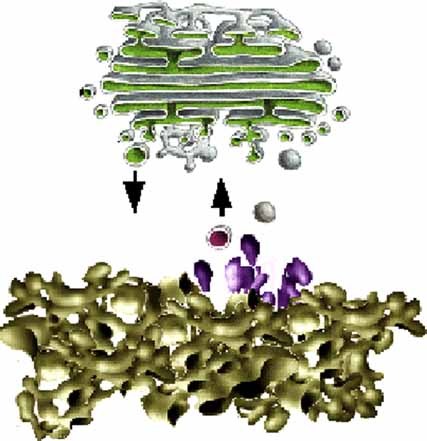
Schematic illustration of fusion and fission processes involving transport processes of vesicles that are trafficking proteins between the endoplasmic reticulum (bottom) and the Golgi apparatus (top). Courtesy of Dr. Matthias Weiss.

Curvature is a key concept in biology 21–24 although in many cases overlooked by the life-science community. Curvature is a so-called emergent property that arises as a consequence of the complex and collective behavior of a large number of molecules. Typically, curvature is a dynamic entity that fluctuates due to entropic forces, and in the case of membranes it varies in time due to transient interactions between the membrane and other cellular components, such as proteins, solutes, as well as vesicles and other membranes. In fact the entire cell, its membranes, organelles, and transport systems are subject to stabilizing and destabilizing forces that couple to curvature. An example is shown in Fig. 1 in the case of intra-cellular trafficking and the dynamical morphogenesis and maintenance of the Golgi apparatus 25.

**Figure 2:**
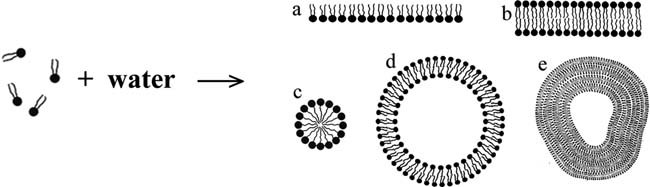
Schematic illustration of the self-assembly process of lipids in water forming aggregates such as (a) a monolayer on the air–water interface, (b) a lipid bilayer, (c) a micelle, (d) a unilamellar liposome (vesicle), and (e) a multi-lamellar liposome.

**Figure 3:**
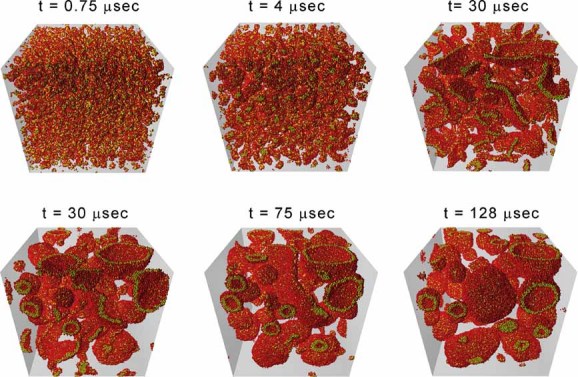
Snapshots from a large-scale computer simulation of the self-assembly process of lipid vesicles (unilamellar liposomes) in water based on dissipative particle dynamics calculations 129. The simulation box is 90 nm^3^ and contains 50.000 lipid molecules in water. The simulation covers a time span of 128 µs. Courtesy of Dr. Julian Shillcock.

Of the four main classes of macromolecules which make up all types of biological systems: the carbohydrates, the fats, the proteins, and the nucleic acids – the fats, e.g., the lipids, distinguish themselves as molecules that in contrast to the three other classes are not polymers bound by covalent forces. Carbohydrates are poly-saccharides, proteins are poly-peptides, and nucleic acids are poly-nucleotides. The lipids do not form polymers under natural conditions, both rather self-assemble into macromolecular aggregates in water in the form of superstructures like micelles and lipid bilayers as illustrated in Fig. 2. Of these structures, the closed lipid bilayer in the form of a unilamellar vesicle or liposome is a structure of fundamental importance for life since it is a model of the lipid-bilayer component of cell membranes 26–31. A detailed visualization of the spontaneous formation of an ensemble of self-assembled liposomes in a suspension of lipid molecules in water is shown in Fig. 3.

It is the self-assembly nature of lipid bilayers that holds the key to both a fundamental understanding of lipid membrane structure, dynamics, and function as well as to many technological applications of lipids. However, lipid self-assembly and the complexity it imparts on lipid behavior is one of the main sources for the difficulties associated with a quantitative description of lipids in biology. A central dogma in molecular biology is molecular structure and the relationship between structure and function. This dogma has been a driving force for the grand achievements of molecular biology during the second part of the 20th century: the structure of the double DNA helix and the structural characterization of the gene products, the proteins. Speaking about structure in this context, it is high-resolution structure with atomic detail that is in focus. Although the importance of molecular dynamics and heterogeneity as well as the influence of solvent structure and dynamics on protein structure and function are acknowledged, it is clear that the unraveling of a well-defined atomic structure has been the Holy Grail in molecular and structural biology.

If one is looking for similar elements of molecular structure and order when it comes to lipids and membranes, one is going to be disappointed. The hallmark of lipids in functional membranes is just as much disorder as it is order. In fact the balance between order and disorder is likely to be at the core of regulation of biological function by lipids. However, disorder and the way order emerges out of disorder is not an easy concept to grasp, and it requires methods from physics and physical chemistry for dealing with in a quantitative manner. This situation has implied that lipids until recent years have been living in the shadow of the more fashionable study of genes and proteins. Lipids were at best described by such fuzzy terms as variability, diversity, plasticity, adaptability, fluidity, complexity, etc., cf. Table 1. These properties, which lipids share with other soft-matter materials, are all of collective and emergent nature and in principle basic consequences of the many-body nature of the lipid aggregates. None of these collective properties are easy to define and measure quantitatively and they are only related to the molecular and chemical structure of the individual lipid species in rather subtle ways. However, as we shall see later, it is exactly those fuzzy attributes to lipids that make lipids so useful for a wide range of technological applications.

**Table 1:** Some unique characteristics of soft-matter systems made of lipids

Soft-matter characteristics of lipid systems
Bottom-up
Self-organized
Self-assembled
Versatile
Diverse
Plastic
Adaptable
Flexible
Complex
Fluid
Length-scale tunable
Durable
Self-repairing
Self-healing

One of the outstanding mysteries in lipid cell biology is lipid diversity, i.e., the fact that each type of membrane has a very large number of different types of lipid species. As information on the lipidome of various cells and subcellular structures is accumulated it is becoming clear, that this diversity is much larger than previously thought, and typically several thousands of different lipids are found to be present in each type of membrane. Considering the many ways all these different species can arrange among themselves, it is clear that the “language” or the “alphabet” of lipids is way beyond the four-letter alphabet of the nucleotides used to describe nuclei acids and the twenty-letter alphabet of the amino acids used to describe proteins. The omics-language of lipids is thus far richer than previously anticipated thereby exposing the full scope of the potential of what Rilfors and Lindblom in 2002 coined “functional lipidomics” 32.

## 2 The powerful language of shape

**Figure 4:**
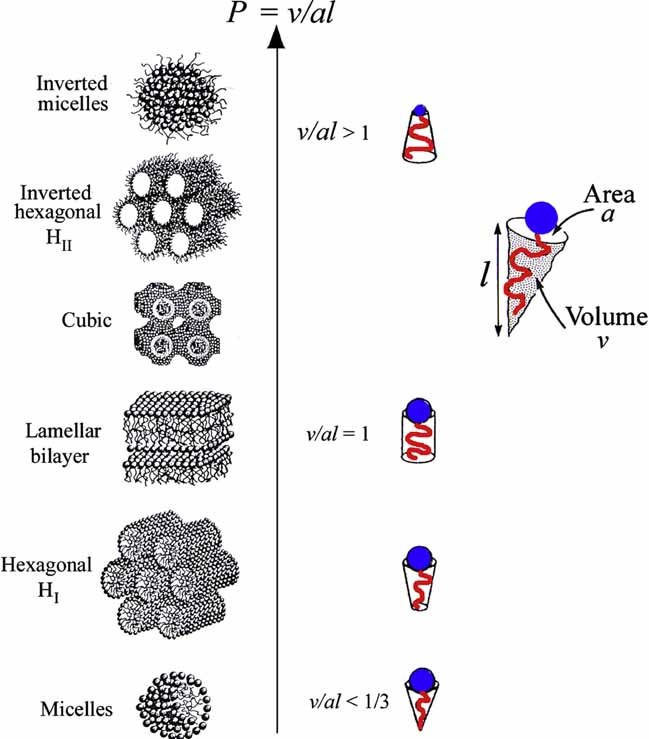
Schematic illustration of lamellar and non-lamellar lipid aggregates formed by self-assembly processes in water. The different structures have different senses of curvature and are arranged in accordance with the value of the phenomenological molecular packing parameter, *P* = *v*/*al*, where *v* is the molecular volume, *a* is the cross-sectional area of the head group, and *l* is the length of the molecule. Adapted from 34.

The various lipid aggregates resulting from lipid self-assembly processes as shown in Fig. 2 are only a few of the many possible ones 33–37. Others are shown in Fig. 4. It is noteworthy that even topologically complex and highly curved structures like hexagonal and cubic phases, as well as a number of more disordered structures, are all found in living cellular systems 21. It should also be noted that even if a particular non-lamellar and curved structure is not found as an extended structure in a cell, it may well be so on a small and local scale, such as in the context of fusion and fission processes, cf. Fig. 1, as well as in the neighborhood of membrane inclusions/proteins or in the form of membrane defects such as pores. More importantly, a membrane may be subject to instabilities due to intrinsic curvature stress and propensity for forming curved structures.

**Figure 5:**
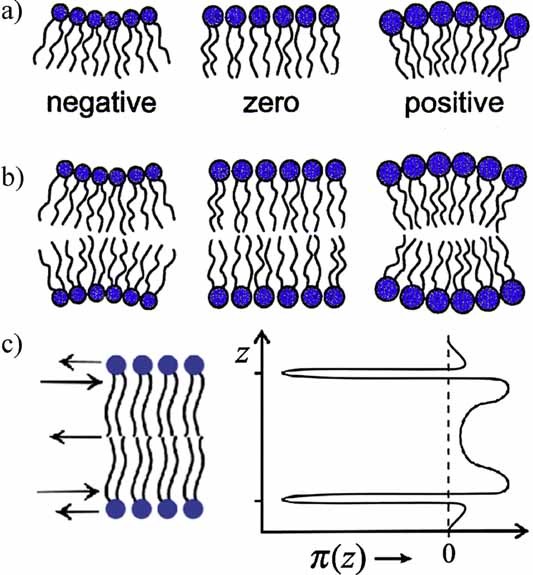
(a) Lipid monolayers with negative, zero, and positive (from left to right) curvature determined by the conicity of the lipid molecules. (b) Stable lipid bilayer (middle) formed by two opposing lipid monolayers. If the monolayers were not constrained by being in the bilayer, they may want to curve as shown to the left and the right, in which case the middle stable bilayer would suffer from a built-in curvature stress. Courtesy of Dr. Olaf Sparre Andersen. (c) Schematic illustration of the lateral pressure profile, π(*z*), of a lipid bilayer, revealing regions of expansive (positive) pressures and regions of large tensile (negative) pressures.

It is possible qualitatively and in some cases semi-quantitatively to describe the lipid phase behavior via a simple geometric property of the lipid molecule, the so-called Israelachvili–Mitchell–Ninham packing parameter, *P* = *v*/*al*, where *v* is the molecular volume, *a* is the cross-sectional area of the head group, and *l* is the length of the molecule 37. Of course a lipid molecule in a dynamic lipid aggregate cannot be assigned a shape as such, and the geometric parameters *v*, *a*, and *l* should therefore be considered as average properties. Still, the value of *P* turns out to be surprisingly useful in predicting the structure of a lipid aggregate. Lipids with values of *P* not too close to unity are poor bilayer formers. However, often the lipids in the two lipid monolayer leaflets of a thermodynamically stable bilayer have, as illustrated in Fig. 5a and b, values of *P* different from unity and hence suffer from a built-in curvature stress. Such monolayers would curve if they were allowed to do so and not being confined to constitute a stable bilayer. In some cases, the effective value of *P* for a mixture of lipids with different values of *P* can be estimated by a superposition principle, provided that the lipid molecules are well mixed. As an example it is possible to form stable bilayers of equimolar mixtures of lysolipids and free fatty acids, that respectively have *P*<1 and *P*>1 38–40. Even in cases where the quantitative predictive power of the molecular packing parameter fails, it can be useful in estimating the influence of a particular molecular species on the stability of a given aggregate structure, e.g., the destabilizing effect of conical molecules added to a lamellar lipid bilayer. The packing parameter turns out to be linearly related to the old concept of hydrophilic-lipophilic balance (HLB) 41.

The self-assembled nature of lipid bilayers implies that they are normally in a tension-less state. The most conspicuous feature of a lipid bilayer is its transverse structure which is far from that of an isotropic fluid slab of hydrocarbons. It displays a distinct lateral stress- or pressure profile 42–45 as illustrated in Fig. 5c. The physics behind this profile is based on simple mechanics. In mechanical equilibrium in the tension-less state, the integral of the difference between the normal pressure and the lateral pressure, *p*_N_(*z*)–*p*_L_(*z*), has to become zero. However, the variation of the lateral pressure across the 5 nm thick membrane goes from positive, expansive pressures in the head group region, over regions of negative, tensile pressures in the interfacial regions, to expansive, positive pressures in the acyl-chain region, as illustrated in Fig. 5c. These variations can easily amount to the equivalent of hundreds of atmospheres pressure. It is this very stressful environment integral membrane proteins have to come to terms with.

**Figure 6:**
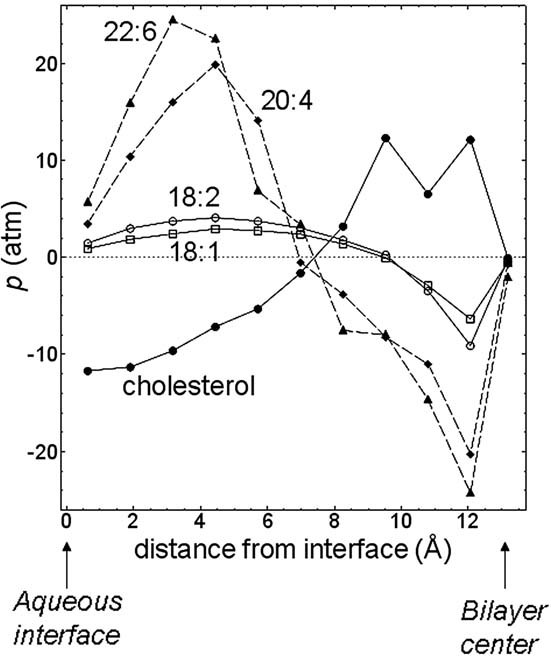
Theoretical prediction of changes in the lateral pressure profile of lipid bilayers that become incorporated with 1 mol% unsaturated lipids of different types into a DPPC lipid bilayer. The data are compared to the opposite changes induced by cholesterol incorporation. Courtesy of Dr. Robert S. Cantor.

In order to illustrate the potential of using molecular shape as a simple means of predicting the effect of various lipid species on the lateral pressure profile of membranes, we consider two types of molecules with different values of *P*: unsaturated lipids which tend to have disordered and curly chain configurations and hence *P*>1, and cholesterol which has a small head group and a bulky hydrophobic body and hence *P*<1. The unsaturated lipids are therefore expected to move the pressures toward the membrane–water interface, and this effect should increase with the degree of unsaturation. In contrast cholesterol is expected to shift the pressures toward the middle of the bilayer 46. The data shown in Fig. 6 indeed supports these expectations 47. These effects imply dramatic changes in the curvature stress of the bilayer and an increase in the flip-flop rate between the two monolayer sheets 48. Possibly even more important, introduction of an extra double bond in the acyl chain, specifically from five to six, has a significant effect on the lateral pressure profile whereas it appears only to have a marginal effect on other bulk bilayer properties 49. Furthermore, the actual position of the double bonds has a significant effect on form of the lateral pressure profile 50. All these findings are of importance for the functioning of neural membranes which are rich in superunsaturated ω-3 and ω-6 fatty acids.

Lipid bilayers and membranes exhibit substantial structure in the plane of the membrane. This has been known for a long time and has been described in terms like lipid domains and lateral heterogeneity 51–54. The lateral structure can be induced by thermodynamically driven phase separation in multi-component systems, lateral density and compositional fluctuations in equilibrium, non-equilibrium and steady-state driven lateral organization in the active state, as well as interactions between lipids and proteins. A particular important mechanism involves cholesterol which invariably is involved in domain organization in particular in plasma membranes 55. Cholesterol is known to be the source of the so-called liquid-ordered state of membranes 56, 57. The interest in the biological importance of lateral membrane heterogeneity and domains has been revived by the so-called raft hypothesis 16, 58–60 that assumes differentiated small-scale regions in biological membranes to be particular platforms for a variety of cell functions, such as signaling and different transport processes 14, 61.

**Figure 7:**
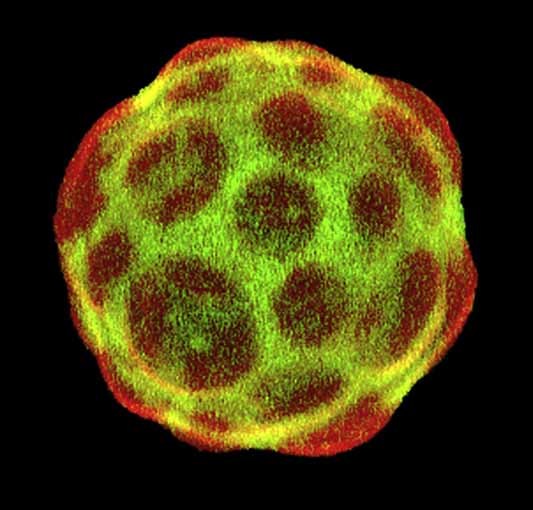
Fluorescence microscopy image of a 17 µm giant unilamellar liposome composed of native pulmonary surfactants from pig lung displaying fluid–fluid phase separation accompanied by cap formation 130. From http://www.scienceinyoureyes.com by courtesy of Drs. Jorge Bernadino de la Serna and Luis A. Bagatolli.

The lateral structure may couple to curvature and enforce the membrane to make both dynamic and static excursions in the third dimension in the form of curved domains that may develop into caps and buds 62–65. An illustration of cap formation on a giant unilamellar liposome is shown in Fig. 7. The cap formation and the associated domain size are controlled by a balance between the bulk free energies of the different regions of the membrane and the line tension around the cap. The finding of finite-size domains in vesicles of lipid mixtures which otherwise would be expected to undergo macroscopic phase separation has also been described theoretically by a balance between the tendency to form macroscopic phases, the line tension of the domains, and a coupling to bilayer curvature 66.

**Figure 8:**
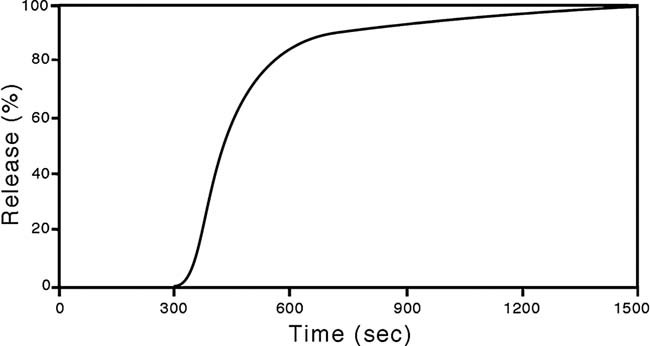
Percentage release as a function of time of doxorubicin from 100 nm liposomes composed of POPC lipids upon addition of 20 µM palmitic acid 67. Courtesy of Dr. Henrik Jespersen.

As an example of the power of lipids with conical shape (*P* ≠ 1) to impact on lipid bilayer properties we show in Fig. 8 how a fatty acid (*P*>1) can lower the permeability barrier of lipid bilayers in the form of a closed liposome encapsulating an anti-cancer drug, doxorubicin 67. When integrating into the lipid bilayer, the fatty acid increases the curvature stress which leads to more leaky membranes. A similar behavior has been found for a wide range of saturated and unsaturated fatty acids as well as lysolipids which have the opposite sense of curvature (*P*<1) 39, 67–73. Lysolipids also promote drug release, although less dramatically, probably because they are more water soluble compared to a fatty acid with the same acyl chain. The details regarding how different fatty acids and lysolipids affect liposomal permeability and how it depends on the structure of the lipid bilayer (phase state, temperature, thickness, etc.) is complex and not fully understood at present. However, as we shall come back to later, these effects can be exploited to release the payload of drugs in liposome-based drug delivery.

## 3 Curvature stress and protein function

**Figure 9:**
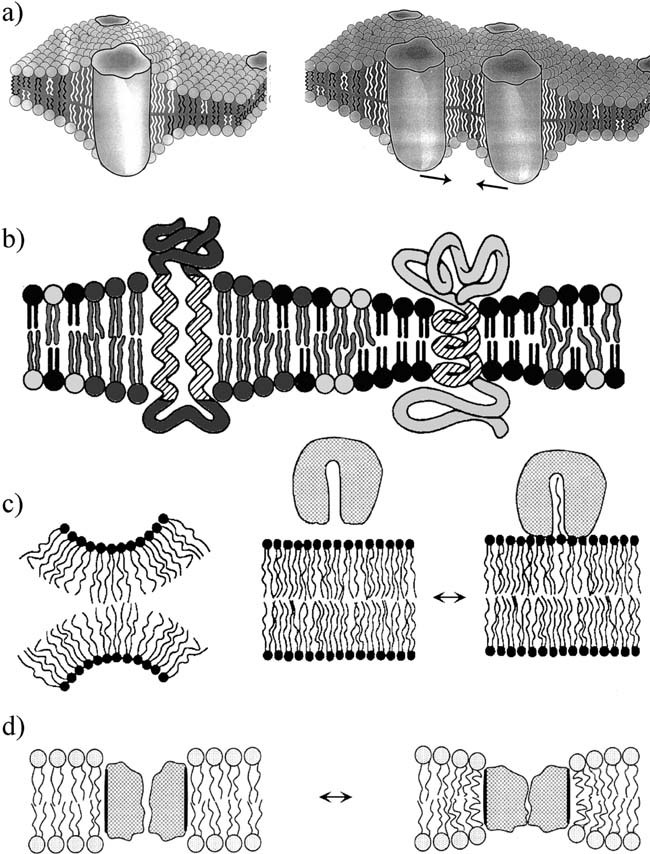
(a) Schematic illustration of the principle of hydrophobic matching between lipid bilayers and integral membrane proteins. In the case of a mismatch, the deformation in the lipid matrix may induce an indirect, lipid-mediated attraction between the proteins. (b) Schematic illustration of a conformational change in an integral membrane protein induced by changes in the hydrophobic mismatch condition (Adapted from ref. 3). (c) Release of the curvature stress in a lipid bilayer, composed of two lipid monolayers with spontaneous curvature, via the formation of the extended lipid chain conformation. One of the tails of the lipid molecule is captured in a hydrophobic pocket of a peripheral membrane protein, e.g., cytochrome c. Courtesy of Dr. P. K. J. Kinnunen. (d) Curvature stress may induce conformational changes in a membrane channel and hence shift the equilibrium between an open and a closed state. Courtesy of Dr. O. S. Andersen.

Proteins associated with membranes, both integral and peripheral proteins, have to come to terms with the curvature stresses in the bilayer 42, 74, 75. In the case of integral membrane proteins, the concept of hydrophobic matching between the hydrophobic core of the lipid bilayer and the hydrophobic stretch of integral membrane proteins has been proposed 76–79 as a key determinant of lipid–protein interactions as illustrated in Fig. 9a. Mismatch carries an energy penalty which basically amounts to the elastic distortion of the lipid matrix around the protein. For a sufficient large value of this penalty, the protein may yield and undergo a conformational change, hence offering a mechanism for lipid-mediated effects on protein function, as illustrated in Fig. 9b. Hydrophobic matching provides in a membrane with several types of lipid species the possibility of sorting, selection, or enrichment of certain lipids near the protein 80, 81.

The hydrophobic matching and the induced membrane curvature around the protein constitutes a mechanism for lipid-mediated protein–protein interactions, typically attractive as illustrated in Fig. 9a, which will provide a driving force for protein aggregation and possibly crystallization in the plane of the membrane. The range of this protein–protein interaction will be controlled by the coherence length of the correlations between the lipids. This coherence length can be very large in special parts of the phase diagram, in particular close to critical points 82, 83, 84, and lead to capillary-condensation phenomena and wetting around proteins 85, 86.

The hydrophobic matching principle has been important in substantiating the concept of membrane rafts 14, which are domains enriched in cholesterol and high-melting lipids, in particular sphingolipids, and therefore generally thicker than the membrane matrix in which they reside. Matching then furnishes a possible mechanism for protein selection where those proteins, which match the raft thickness the better, e.g., via appropriate acylation or prenylation, are recruited to the raft. Conversely they can be released from the raft by appropriate enzymatic modification of the proteins. Hence, the concept of hydrophobic matching helps to establish a semi-quantitative physical framework for signaling cascades.

The propensity of some lipids for inducing curvature stress and possibly non-lamellar phases provides, via the lateral pressure profile, another mechanism for lipid–protein interactions 45, 87 as illustrated in Fig. 9b. This mechanism is not necessarily independent of the hydrophobic matching mechanism. An illustration of this curvature-driven mechanism is given in Fig. 9c and d respectively in the case of release of curvature stress by binding a peripheral membrane protein, such as cytochrome c 88, and a shift between two conformations of a membrane channel, such as the opening and closing of gramicidin A dimer channels 79. Certain proteins, e.g., the nicotinic acetylcholine receptor and the voltage-dependent potassium channel may also have conical shape 22 and they therefore tend to arrange themselves with lipids that match this shape in order to maintain the lamellar membrane structure. It is interesting to note that the total lipid extract of many plasma membranes do not form lamellar phases when suspended in water solution indicating that proteins and lipids exhibit some kind of shape compensation in intact biological membranes.

Most of the quantitative, fundamental insight into membrane structure, dynamics, and molecular organization has been obtained from various model studies, experimentally as well as theoretically, and they almost invariably refer to systems in or near thermodynamic equilibrium 31. However, functioning biological membranes are not in equilibrium but are constantly subject to exchange of energy and material with the environment or are being modulated by active proteins and enzymes that are associated with the membranes 89. This association often involves coupling between the protein and the membrane curvature or the stress fields of the lipid bilayer. It is well known from statistical physics that the properties of non-equilibrium systems are fundamentally different from their equilibrium counterparts, e.g., new levels of organization arise in driven-diffusive systems, and order may emerge out of disorder due to an external drive 90.

A quantitative study of the physical properties of model membranes out of equilibrium is extremely difficult. Hence the results of only few studies have been published 91–100. A typical example is a membrane with an ion pump that is driven by some kind of energy transduction mechanism 96, 101. Another example is the binding of ligands to receptors where the binding is influenced by a force, e.g., the binding of collectins in the innate immune system to sugar groups on invading pathogens 102. A third example is the set of responsive membranes in the dermal barrier that is subject to a gradient in water chemical potential 103. A fourth example is the morphogenesis of the endoplasmatic reticulum and the Golgi apparatus with membranes that owe their existence to non-equilibrium conditions of flow of energy and matter 25. It is interesting in this context to note that the plasma membrane can undergo phase separation when the cell dies and the activity comes to a stop 14.

## 4 Peripheral proteins and enzymes that sense curvature

**Figure 10:**
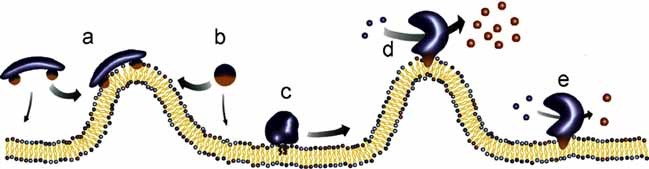
Schematic illustration of proteins and peptides that sense membrane curvature and lipid packing. (a) Bar domain that bind to membrane regions with high curvature. (b) Amphiphatic helix that respond to lipid packing. (c) Protein with hydrophobic anchors that sense lipid packing. (d) and (e) Enzymes, such as protein kinase C or PLA_2_, with high activity at curved membranes and lower activity at planar membranes. Courtesy of Dr. D. Stamou, reproduced with a permission of the publisher 106.

A range of important biological functions mediated by membranes appear to be partly or fully controlled by local membrane curvature 23. Examples include membrane-curvature control of dynamin polymerization 104, phosphocholine cytidylyltransferase activity 105, and the binding of bar domains to membrane surfaces 22, 23, 106, 107 as illustrated in Fig. 10. This figure also indicates that the action of many enzymes and drugs, e.g., amphiphatic and antibiotic peptides 107, 108, is mediated by coupling to membrane curvature which in some cases induces morphological alterations at and in the membrane.

**Figure 11:**
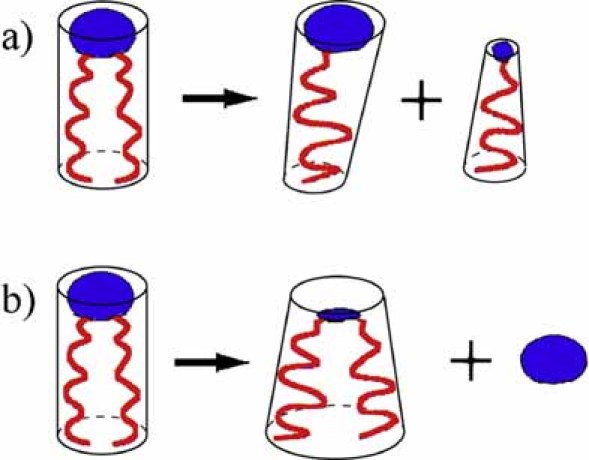
Formation of conically shaped molecules by enzymatic action. (a) PLA_2_ generates lysolipids and free fatty acids. (b) Phospholipase C generates di-acylglycerol by chopping off the head group of the lipid.

A particular class of enzymes acts on membranes by degradating phospholipids or sphingolipids, e.g., phospholipase A_2_ (PLA_2_), phospholipase C, and sphingomyelinase. These enzymes remodel the membranes by producing products that may have propensity for forming non-lamellar, curved lipid structures. Specifically PLA_2_ generates lysolipids and free fatty acids, and phospholipase C generates di-acyl glycerol, as illustrated schematically in Fig. 11. Sphingomyelinase produces ceramide which is known to lead to pronounced membrane curvature and blebbing, a phenomenon that has been associated with apoptosis 109.

**Figure 12:**
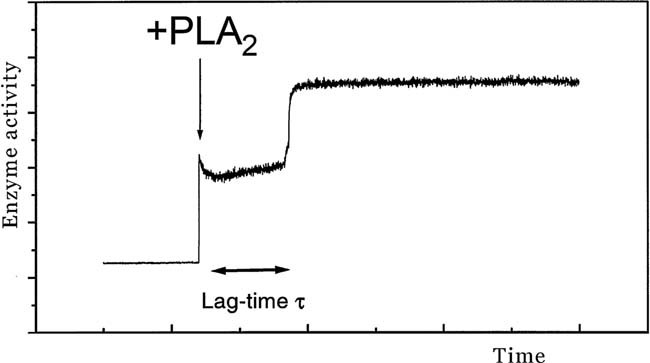
Lag-burst behavior of s-PLA_2_ acting on a lipid bilayer substrate. Upon addition of the enzyme a characteristic lag period follows during which very little activity can be discerned. After a lag-time τ a sudden burst of activity sets in. The activity can be monitored, e.g., by detection of intrinsic tryptophan fluorescence.

Extensive studies have been carried out in order to unravel the mechanism of secretory phospholipase A_2_ (s-PLA_2_) action on lipid bilayers of different composition and under different physico-chemical conditions 110, 111, 112. s-PLA_2_ is only active at lipid interfaces and not on lipid monomers in solution. Moreover, it turns out that its enzymology is rather peculiar in the sense that the enzyme displays a so-called lag-burst behavior, as illustrated in Fig. 12. The lag-time turns out to be extremely sensitive to the physical state of the bilayer substrate, in particular its lateral heterogeneity and defect structure. The more defects and heterogeneity, the shorter the lag-time. The heterogeneities in turn can be controlled by a long list of factors, including temperature, composition, phase transitions, phase separations, compositional and density fluctuations, as well as edge effects. The structural heterogeneity can be seen as a kind of local defects with high curvature and associated with enhanced lipid protrusion modes 113, 114 that will trigger the enzyme activity. The heterogeneity is further enhanced by the hydrolysis products that lead to increased curvature stress and local defects in the bilayer. In this way the enzyme autocatalyzes its own activity which eventually leads to the burst in activity.

The important lesson from these studies is that s-PLA_2_ is extremely sensitive to the structure and physical properties of the lipid bilayer substrate, in particular local curvature and defects. The enzymatic action leads to a remodeling of the membrane 112. This observation can be exploited when constructing liposomal drug-delivery systems tailored to be sensitive to enzymatic breakdown and subsequent localized drug release in cancerous tissue which is characterized by elevated levels of s-PLA_2_. We shall address this in further detail below.

A particular way of activating s-PLA_2_ on liposomal surfaces was somewhat surprisingly discovered in studies of the activity on lipid bilayers incorporated with lipopolymers which are lipids to whose head group is covalently linked a water-soluble polymer, typically poly-ethylene-glycol (PEG). It turns out that s-PLA_2_ is more active on such surfaces compared to naked lipid bilayers. Furthermore, the activity is stronger, i.e., the lag-time is shorter, the larger the surface coverage of the polymers is and the longer the polymer chains are 115. The explanation of this phenomenon is an entropic pull on the lipopolymers which tends to enhance the protrusion modes of the neighboring lipid molecules rendering them more prone for attack of the lipase. The entropic pull is caused by the confinement of the water-soluble PEG chain which cannot penetrate the membrane leading to a decrease in the conformational entropy of the chain. To compensate for the corresponding loss in free energy, the lipopolymer tends to be displaced somewhat into the aqueous compartment 113. Hence, the physico-chemical properties of the PEGylated lipids offer themselves as another control parameter for regulation of the enzyme activity.

## 5 Liposomal drug-delivery systems with enzymatic triggering via curvature stress

We will now show how it is possible to combine the insight in curvature stress and the physical chemistry of lipid bilayers with an understanding of the mechanism of activating s-PLA_2_ in order to construct a liposomal drug-delivery system that may remove a critical bottleneck in the use of liposomes for cancer therapy. The resulting delivery system is hence constructed on the basis of fundamental principles of physical chemistry and physical enzymology. This approach to drug research, which already has been subject to phase-I clinical trails, is unconventional in the sense that it is predominantly based on physical sciences and the concept of membrane curvature stress rather than medicinal chemistry and traditional drug-receptor considerations.

The proposed system takes advantage of the specific biophysical properties of the lipid bilayer 116, 117 of liposomes on the one side and the peculiar pathophysiological and biochemical properties of cancer cells on the other side. Thereby it becomes possible to passively target liposomes to diseased cells and with a particular mechanism, involving endogenously upregulated s-PLA_2_, to open the liposomal carriers and unload the drug precisely where the drug is supposed to act. It is furthermore possible to construct the liposomes of specific lipids that upon phospholipase-assisted hydrolysis are turned into products that may act as enhances, as permeabilizers, and even as drugs themselves 118–120.

**Figure 13:**
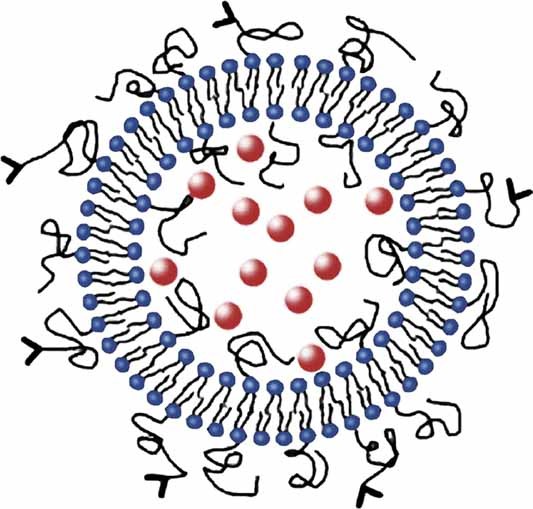
Schematic illustration of a stealth liposome with a polymer coat of PEG that screens the liposome from the native immune system in the blood stream.

Liposomes for drug delivery are usually protected from the human immune system by a polymer coat made up of PEG moieties that are covalently linked to the head group of charged lipids, such as DSPE or DSPS, cf. Fig. 13. This type of PEGylation, which is the basis of the so-called stealth liposomes 121, has two important consequences. Firstly, it helps to avoid release of the encapsulated drug upon intravenous administration in the blood stream, hence limiting the systemic side effects of the drug. Secondly, it leads to increased circulation lifetimes and hence enhances the likelihood for liposomes of appropriate size, typically 100 nm, to venture into the capillary network of, e.g., solid tumors. The tumor vasculature is often rather fenestered and since the lymphatic drainage of the tumors is suppressed in comparison with that in healthy tissue, liposomes tend to accumulate in the tumor. This effect is called the enhanced permeability and retention (EPR) effect.

It has been known for some time that liposomal drug-delivery systems can limit systemic side effects and one of the successful formulations is Doxil 122 which are stealth liposomes with doxorubicin approved for treating, e.g., ovarian cancer. A paradoxical limitation of the efficacy of the liposomes including Doxil is that their stability turns into a disadvantage at the target where the drug has to be unloaded and in many cases the unloading of the drug is very poor. Hence an effective trigger mechanism for discharging the payload is called for.

One such trigger mechanism is the use of heating the tumor in the case where the liposomes have been made thermosensitive, e.g., by having a phase transition that is a few degrees above physiological temperatures 123, 124. At the phase transition, the lipid bilayer of the liposomes becomes leaky 8 and the drug can escape into the tumor tissue. The thermal triggering mechanism requires that the tumor can be localized and heated by external heating devices. A system of this type is currently in phase-III clinical trials for treatment of liver cancer 124.

Another possible trigger mechanism is the use of endogenously upregulated s-PLA_2_ to destroy the liposomes at target. In turns out that many different kinds of tumors, e.g., breast, colon, gastric, pancreas, lung, liver, and prostate cancer, have such upregulated type IIA s-PLA_2_ in a local concentration in the tumor that is one or two orders of magnitude larger than serum levels and much higher than in the healthy tissue lining the tumor 125–127.

Stealth liposomes that have been made sensitive to hydrolysis by s-PLA_2_ have been coined LiPlasomes. These lipase-labile liposomes have been proposed as suitable carriers for drugs targeted to tumors with locally high levels of s-PLA_2_ activity 20, 117. The development and design of the LiPlasomes is based on years of extensive studies of lipid bilayers and model membranes and how the activity of s-PLA_2_ on these lipid systems can be controlled by the physical properties and qualities of the lipid bilayers 110.

**Figure 14:**
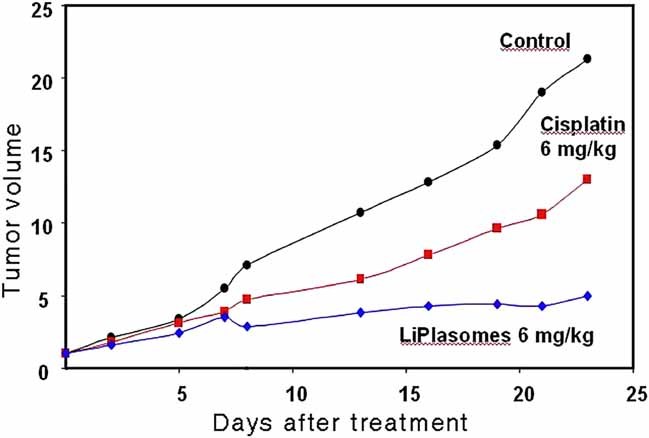
In vivo proof of principle of the efficacy of LiPlasomes loaded with cisplatin applied to a human xenograft MT-3 breast cancer in mice. Data from LiPlasome Pharma A/S.

LiPlasomes have been tested on a variety of in vitro cell cultures of cancer cell lines that secrete s-PLA_2_ 20 with encapsulated anti-cancer drugs like doxorubicin, cisplatin, and 5-FU (fluorouracil), and in all cases it has been found that LiPlasomes poised to unload the drugs by s-PLA_2_ action limit the cell growth whereas conventional liposomes are much less effective. The crucial next step involves in vivo studies on mice, and Fig. 14 shows as an example that cisplatin-loaded LiPlasomes are very effective to limit the growth of human xenograft MT-3 breast cancer in mice. This encouraging in vivo proof-of-principle was the basis for bringing the first dose of LiPlasomes in man.

It is noteworthy from Fig. 14 that the mice treated with LiPlasome formulations with cisplatin are not only doing much better than the control group; in fact they do better than the group of mice treated with the free drug at similar toxic doses.

A plausible reason for this remarkable effect becomes clear when considering the action of s-PLA_2_ on the LiPlasomal carrier. The enzyme not only opens the capsule by hydrolyzing the lipids. It also releases the hydrolysis products, lysolipids and free fatty acids, as illustrated in Fig. 11a. As we described above and illustrated in Fig. 8 these products act as enhancers and permeabilizers because they induce curvature stress in the target membranes of the cancer cells, thereby lowering the permeability barrier and facilitating the transport of the active drugs into the cells. In this way the phospholipids of the liposomal carrier have acted not only as materials for the liposome; they have also performed as pro-enhancers and pro-permeabilizers that are turned into enhancers and permeabilizers exactly where they are needed.

## 6 A futuristic scenario in nano-medicine: Lipid prodrugs

Once it has been established that it is possible to construct LiPlasomes which are stable in the blood stream, which accumulates in the diseased tissue, and whose drug load can be released by endogenously upregulated s-PLA_2_; and once it has been established that the hydrolysis products can act as enhancers and permeabilizers; a scenario suggests itself by which the lipids in the liposomal carrier themselves are prodrugs which by s-PLA_2_ action are turned into active drugs precisely at the target. In principle, by this scenario the LiPlasomes could be empty and seen as a magic bullet, the LiPlasome acting at the same time both as a carrier and as a drug. Needless to say, such LiPlasomes could also be loaded with conventional drugs such as doxorubicin or cisplatin to be used in a special type of combination therapy.

The first type of prodrug lipids that come to mind are mono-ether-mono-ester glycerol-phospholipids, in which the *sn*-1 chain is linked to the glycerol backbone by an ether linkage and the *sn*-2 chain by an ester linkage 128. Upon s-PLA_2_ action, a lysoetherlipid and a free fatty acid are released. Certain types of lysoetherlipids are known to be strongly cytotoxic, but since the red blood cells lack enzymes to degradate these etherlipids, injection of such compounds in the blood stream leads to massive hemolysis. Cancer cells are also not able to break down the etherlipids and their membranes will be massively damaged by the compounds. Hence a unique possibility opens for using lysoetherlipids to treat cancer by tugging the hemolytic etherlipid away in a mono-ether-mono-ester phospholipid compound. As long as the etherlipid remains bound in this compound it turns out not to be hemolytic 128. However, once it is released by s-PLA_2_ action in the tumor tissue it can act. The feasibility of this mechanism has been demonstrated by in vitro studies on gastric cancer cell cultures that secrete s-PLA_2_.

We are now ready to make a further step and consider also making the entity linked to the phospholipid on the *sn*-2 position a potential drug, i.e., a prodrug that is turned into an active drug upon release by s-PLA_2_ action in the cancer tissue. This new type of phospholipid consisting of an lysoetherlipid and another active lipid drug substance can be considered a double lipid prodrug.

**Figure 15:**
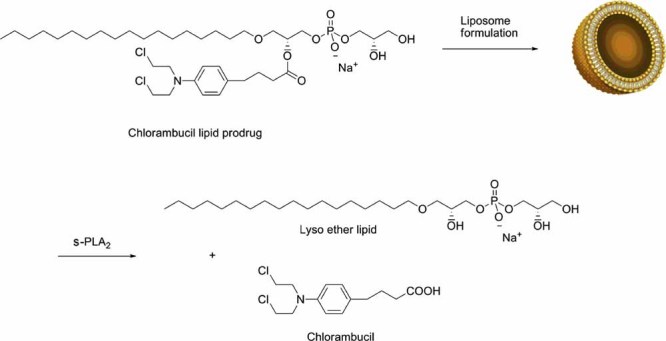
Liposomal formulation based on a double lipid prodrug with the anti-cancer drug chlorambucil ester-linked in the *sn*-2 position and where the *sn*-1 chain is linked by an ether bond. Upon the action of s-PLA_2_ the two prodrugs are turned into active drugs. Adapted from 118 with a permission of the publisher.

We have successfully synthesized a series of such double lipid prodrugs, with an etherlipid at the *sn*-1 position and potent drugs like chlorambucil 118 or derivatives of retinoic acid 119, 120 on the *sn*-2 position. An illustration of this double lipid prodrug principle in a liposomal formulation is provided in Fig. 15. The efficacy of these lipid prodrug systems has been evaluated in a number of different in vitro cell culture studies of various human cancer cell lines and it is found that the combination of the two lipid drugs enhances the cytotoxicity.

The further development of such double lipid prodrug systems requires optimization with respect to choice of both the actual active chemical components that are built into the lipids and the other lipid constituents of the liposome. Not all constructs will lead to stable liposomes. However, it is possible to secure liposome stability and optimize susceptibility to s-PLA_2_ action by premixing with other lipid species that do not act as drugs but facilitate the liposome formation and possibly even enhances the enzymatic breakdown of prodrugs 120. In this way a dilution of the prodrug may be amply outbalanced by a more effective turnover of the prodrugs into drugs. Finally, combination formulations including an encapsulated conventional drug should also be considered.

## 7 Concluding remarks

The concepts of lipid shape and packing, membrane curvature and curvature stress, as well as the lateral stress profile of lipid bilayers are not new concepts but their potential for use in developing novel types of nano-medicine should not be neglected. Even if these concepts in some cases are less well defined and possibly even difficult to measure and describe quantitatively they can serve as valuable guides for shaping the intuition that is necessary to mobilize for innovation in the treatment of serious diseases.

The present mini-review has illustrated how an insight into the basic physical chemistry of lipids, including their many seemingly fuzzy characteristics listed in Table 1, together with an understanding of the peculiar enzymology of interfacially activated phospholipases may provide a basis for a rational approach to liposome-based nano-medicine.

## Abbreviations

PEG: poly-ethylene-glycol
PLA_2_: phospholipase A_2_
s-PLA_2_: secretory phospholipase A_2_
